# Empire Apple (*Malus domestica*) Juice, Pomace, and Pulp Modulate Intestinal Functionality, Morphology, and Bacterial Populations In Vivo (*Gallus gallus*)

**DOI:** 10.3390/nu14234955

**Published:** 2022-11-22

**Authors:** Cydney Jackson, Viral Shukla, Nikolai Kolba, Nikita Agarwal, Olga I. Padilla-Zakour, Elad Tako

**Affiliations:** Department of Food Science, Cornell University, Ithaca, NY 14850, USA

**Keywords:** apples, intra amniotic administration, gene expression, microbiome, intestinal morphology, *Gallus gallus*

## Abstract

Approximately $20 billion of apple sales are generated annually in the United States. With an estimated 5 million tons produced yearly in the U.S. within the last decade, apple consumption is considered ubiquitous. Apples are comprised of bioactive constituents such as phytochemicals and prebiotics that may potentiate intestinal health and the gut microbiome. This study aimed to evaluate the effects of Empire apple juice, pomace, and pulp soluble extracts on intestinal functionality, morphology, and the microbiome in vivo (*Gallus gallus*). There were five treatment groups: non-injected (NI); 18 MΩ H_2_O (H_2_O); 6% apple juice (AJ); 6% apple pomace (APo); 6% apple pulp (APu). The eggs were treated by intra-amniotic administration of the samples on day 17 of incubation. After hatching, the blood, tissue, and cecum samples were collected for further analyses—including duodenal histomorphology, hepatic and duodenal mRNA expression, and cecal bacterial populations. Crypt depth was significantly (*p* < 0.5) shortest in AJ when compared to APo and APu. APo and APu soluble extracts significantly improved villi surface area compared to NI and H_2_O control groups. The highest count of Paneth cells per crypt was observed in APo as compared to all groups. In addition, the expression of brush border membrane micronutrient metabolism and functional proteins varied between treatments. Lastly, *Lactobacillus* cecal microbial populations increased significantly in the AJ group, while AJ, APu, and APu increased the abundance of *Clostridium* (*p* < 0.5). Ultimately, these results indicate the potential of Empire apple pomace to improve host intestinal health and the gut microbiome.

## 1. Introduction

Apples (*Malus domestica*) are a well-established, domesticated fruit worldwide, with approximately 86 million metric tons produced annually [1]. On a fresh basis, apples are among the top fruit varieties grown in the United States—second only to grapes [2]. Fresh apple consumption is most common among consumers, however, 35% of the apples consumed are processed [3]. While processed apple products generally include jams, jellies, cider, vinegar, and dried products, most apples are processed for apple juice. There is a 75% juice extraction efficiency in the apple juice industry; thus, 25–30% of the fruit waste remains. Known as the pomace, this leftover fraction is a heterogeneous mixture of skin, flesh, seeds, stems, core, and calyx [4,5]. According to the U.S. Apple Association, approximately 33.4 million bushels (701.4 million kg) of apples were produced in 2021–2022 for juice and cider production [6]. Based on the juice extraction efficiency (75%), an estimated 175,350 metric tons of apple pomace were produced annually [4]. Apple waste is typically disposed of in landfills, leading to several damaging environmental effects, as disruption to the carbon:nitrogen ratio of soil can occur due to sugar content, organic acids, and microbial fermentation of apple pomace [7]. The high-water content of apple pomace is also an issue as it can cause water pollution. Given the complications of pomace disposal, various industries have taken advantage of the by-product as it is a rich source of nutrients such as carbohydrates, micronutrients, and phytochemicals [8]. Further utilization includes pectin extraction, production of enzymes and aroma compounds, cultivation of microbial strains and edible mushrooms, and incorporation into animal feed [5]. However, the burden remains to prevent the disposal of apple waste into landfills and, ultimately, avoid environmental pollution.

Apples contain health-promoting bioactive constituents. Quercetin derivatives (galactoside, glucoside, rhamnoside), catechin, gallic acid, phloretin, and chlorogenic acid are polyphenolic compounds that are reportedly found in apples [8]. Studies have reported the antioxidative [9,10,11,12,13], antiproliferative [11,14,15,16,17], and anti-inflammatory [18,19,20] potential of apple polyphenols. It is important to note that apple phenolic compounds differ in concentration throughout the fruit matrix. For example, while apple flesh and apple peels contain chlorogenic acid, the flesh contains higher concentrations [21,22]. Research has also shown apple peels to contain greater amounts of antioxidants and, therefore, greater antioxidant potential compared to apple flesh. In addition, interest in apple seeds as a source of polyphenolic compounds has been investigated and found to be a rich source of quercetin derivatives, phenolic acids, catechin, and phloridzin [23]. Another constituent with health-promoting properties within the apple is dietary fiber, specifically, the non-digestible soluble polysaccharide pectin. Historically utilized as a commercial thickening and gelling agent, pectin holds potential functional properties which may improve intestinal health. The resistance to gastric digestion enables pectin to reach the host gut and undergo fermentation by microbiota, and ultimately produces short-chain fatty acid (SCFA) metabolites [24,25,26,27]. Previous studies have shown SCFAs to be beneficial to the gut by promoting enteric epithelial cell proliferation, enhancing gut barrier function, enhancing micronutrient absorption, and favoring the growth of beneficial bacteria over potentially pathogenic bacteria [28,29,30,31,32].

This study aimed to evaluate the in vivo effects of apple juice, pomace, and pulp soluble extracts on intestinal morphology, functionality, and the microbiome using *Gallus gallus* as our model. The broiler chicken is an established model employed to evaluate the effects of plant-origin bioactives on intestinal health and the microbiome [33]. The broiler chicken model exhibits genetic homology, a complex gut microbiota, and notable microbial similarity at the phylum level to human gut microbiota [34].

## 2. Materials and Methods

### 2.1. Apple Preparation

Empire apples (*Malus domestica*) were harvested (>2 tons) during the fall of 2021 from multiple trees from the Cornell AgriTech Orchards and processed at the Cornell Food Venture Center Pilot Plant (Geneva, NY, USA). Before processing, Empire apples were destemmed and washed. Apple pulp was made by removing the core and seeds and dicing. The apple pieces were then freeze-dried (Max53, Millrock Technology, Kingston, NY, USA) for 24 h. The dried apple was then ground into a fine powder using a bench-scale processor (Robo Coue; Jackson, MI, USA) at 1500 RPM. Apple juice was made to typical industry standards. For apple juice, whole apples were ground in a hammer mill in a blade configuration. The pulp was then pressed using a pilot-scale hydraulic press (Orchard Equipment Co., Conway, MA, USA) at 1200–1400 PSI. The juice was commercially sterilized by hot-packing into PET bottles at 85 °C and keeping it hot for 2 min. Apple pomace was the resulting pomace from the juice pressing. After juice pressing, the pomace was freeze-dried for 24 h. The dried pomace was ground into a fine powder using a bench-scale grinder. The powders were vacuumed-sealed, and all samples were kept frozen until use.

### 2.2. Apple Analysis Sample Preparation

Apple samples were extracted under dark conditions utilizing absolute methanol and constant agitation for 2 h. The resulting slurry was centrifuged and decanted to acquire the supernatant. The subsequent isolate and washings were diluted to attain an extract (15% *w*/*v*) which was ultimately utilized for the further analysis below.

#### 2.2.1. Polyphenol Analysis

The Folin-Ciocalteu method previously detailed by Waterhouse was utilized to quantify total polyphenol content (TPC) [35]. Essentially, the Folin-Ciocalteu reagent and the extract were allowed to incubate at room temperature. The sodium carbonate solution was used to quench the reaction and sample absorbance was measured immediately using a UV-visible spectrophotometer (Thermo Fisher; Waltham, MA, USA) at 765 nm. Therefore, TPC was calculated as gallic equivalents (GE) using a standard curve prepared under the same conditions.

#### 2.2.2. Fibrous and Non-Fibrous Carbohydrate Analysis

According to AOAC 962.09, the non-fibrous carbohydrate analysis (NFC) was completed. Acid detergent fiber (ADF) and neutral detergent fiber (NDF) analyses were conducted according to AOAC 973.18. The analysis was performed by Dairy One Co-Op Inc. (Ithaca, NY, USA).

### 2.3. Extraction of Soluble Apple Contents

Apple powders and juice samples were dissolved and diluted in distilled water to create 6% concentrations. All apple samples were heated via water bath for 1 h at 60 °C, centrifuged (3500 RPM) for 10 min at room temperature, and the supernatant was collected only in the case of the pomace and pulp.

### 2.4. Animals and Design

Fertile Cornish-cross broiler chicken eggs (*n* = 55) were provided by a hatchery (Moyer’s chicks, Quakertown, PA, USA). All animal protocols were approved by Cornell University Institutional Animal Care and Use Committee (ethic approval code: 2020-0077). Apple extract powders and juice were diluted with 18 MΩ H_2_O to acquire the necessary concentration to maintain an osmolarity value of less than 320 Osm. On day 17 of embryonic incubation, viable eggs were weighed and randomly allocated into five groups (*n* = 12) with a similar weight frequency distribution. After identifying the amniotic fluid by candling, the treatment solution (1 mL) was injected with a 21-gauge needle. Subsequent to injection, the injection site was sealed with cellophane tape. Eggs were placed in hatching baskets for each treatment and equal representation at each incubator location. The five treatment groups consisted as follows: non-injected (NI); 18 MΩ H_2_O (H_2_O); 6% apple juice (AJ); 6% apple pomace (APo); and 6% apple pulp (APu). On day 21, exposure to CO_2_ was used to euthanize hatchlings, and the blood, pectoral muscle, liver, duodenum, and cecum were collected for further analysis.

### 2.5. Blood Analysis

Blood was collected from the heart using micro-hematocrit heparinized capillary tubes (Fisher Scientific Waltham, MA, USA). Blood glucose concentrations were determined using the Accu-Chek^®^ blood glucose monitor following the manufacturer’s protocol. 

### 2.6. Pectoral Glycogen

The pectoral muscle was collected on the day of the hatch. Glycogen analysis was completed as previously described [36,37,38]. Briefly, 20 mg of the sample was homogenized in 8% perchloric acid and centrifuged at 12,000 rpm (4 °C) for 15 min. The supernatant was removed, and 1.0 mL of petroleum ether was added. Following mixing, the petroleum ether fraction was discarded, and the remaining sample layer was transferred to a new container with the color reagent (300 µL). Samples were read in an ELISA reader at 450 nm, and glycogen content was analyzed based on the standard curve. Total glycogen content in the pectoral sample was identified as the product of multiplying tissue weight by the amount of glycogen per 1 g of wet tissue.

### 2.7. Total RNA Extraction from Duodenum and Liver Tissue Samples

Total RNA was extracted from 30 mg of duodenal and liver tissue samples (*n* = 5) as previously described [39,40,41,42,43]. Briefly, the Qiagen Rneasy Mini Kit (Rneasy Mini Kit, Qiagen Inc., Valencia, CA, USA) was used. All protocols were carried out according to the manufacturer and under Rnase-free conditions. RNA was quantified by absorbance at A 260/280. The integrity of 18S ribosomal rRNA was verified by 1.5% agarose gel electrophoresis, followed by ethidium bromide staining. RNA was stored at −80 °C until further use.

### 2.8. Real-Time Polymerase Chain Reaction (RT-PCR)

cDNA was created from the extracted RNA by a 20 µL reverse transcriptase (RT) reaction. To complete the reaction, the BioRad C1000 touch thermocycler using the Improm-II Reverse Transcriptase Kit (Catalog #A1250; Promega, Madison, WI, USA) was utilized. The cDNA concentration was measured by Nanodrop (Thermo Fisher Scientific, Waltham, MA, USA) at an absorbance of 260 nm and 280 nm using an extinction coefficient of 33 (for single-stranded DNA). Genomic DNA contamination was assessed by a real-time RT-PCR assay for the reference gene samples.

The RT-PCR primers were designed based on relevant gene sequences from the GenBank database using the Real-Time Primer Design Tool software (IDT DNA, Coralvilla, IA, USA), as detailed previously [44]. Table 1 indicates the primer sequences used in accordance with iron, zinc, and vitamin A metabolism, immune response, and brush border membrane functionality. The reference gene used was the *Gallus gallus* primer 18S rRNA. BLAST searches against the genomic National Center for Biotechnology Information (NCBI) database were applied to verify primer specificity.

### 2.9. Microbial Samples and Intestinal Contents DNA Isolation

Cecum samples were weighed and placed in sterile tubes containing PBS. Subsequently, the samples were vortexed with sterile glass beads for 3 min. All protocols were completed as previously described [42,45,46,47,48,49].

### 2.10. Primer Design and PCR Amplification of Bacterial 16S rRNA

*Bifidobacterium*, *Lactobacillus*, *Escherichia coli*, *Clostridium*, *Klebsiella*, and *L. plantarum* primers were used. 16S rRNA was the universal primer and internal standard. Therefore, the proportions of each bacterial group are presented. PCR products were applied to 1.5% agarose gel with ethidium bromide stain and quantified with Gel-Pro analyzer version 3.0 (Media Cybernetics LP, Rockville, MD, USA).

### 2.11. Histomorphological Examination

On the day of the hatch, proximal duodenal samples were collected. Subsequently, the samples were soaked in 4% (*v*/*v*) buffered formaldehyde, dehydrated, cleared, and embedded in paraffin. Several sections were cut with a 5 µm thickness and placed on glass slides. Intestinal sections were then deparaffinized in xylene and rehydrated in a series of graded alcohol. Ultimately, the slides were stained with Alcian Blue/Periodic acid-Schiff and investigated by light microscopy using EPIX XCAP software (Standard version, Olympus, Waltham, MA, USA). The following features were measured in the duodenum: villus surface area, crypt depth, villus and crypt goblet diameter, crypt goblet cell number and type, and Paneth cell number and diameter within the crypt, as previously described [42,43,49,50,51]. Per treatment group, five biological samples (*n* = 5) (four segments each) were analyzed. Ten randomly selected villi and crypts were measured and analyzed, and cell measurements and counts were completed in ten randomly selected villi or crypts per segment. The following equation was utilized to calculate the villus surface area:

(1) $$Villus\;surface\;area=2\pi \times \frac{VW}{2}\times VL$$

in which *VW* is the mean of three villus width measurements, and *VL* is the villus length.

### 2.12. Statistical Analysis

In this paper, values are portrayed as the mean values ± standard error means. Experimental treatments and controls for the intra-amniotic administration were assigned with approximately equal weight distribution. Tested parameters were analyzed for normal distribution and equal variance through a Shapiro-Wilk test. If the test was accepted, a one-way analysis for variance (ANOVA) was utilized. ANOVA was used to analyze the results, followed by a Duncan post-hoc test to determine significance based on *p*-values (*p* < 0.05). For all statistical evaluations, software SPSS version 20.0 was utilized.

## 3. Results

### 3.1. Apple Polyphenol and Fiber Content

Empire apple pulp had the highest (*p* < 0.05) TPC content, followed by apple pomace (APo) and apple juice (AJ) (Table 2). Apple pomace had the greatest acid detergent fiber (ADF) and neutral detergent fiber (NDF) compared to apple pulp and apple juice. Lastly, non-fiber carbohydrate content was greatest in the apple pulp. 

### 3.2. Body Weight, Blood Glucose, and Glycogen Content

No significant differences were observed between the treatment and control groups for body weight, blood glucose, and glycogen content (*p* < 0.05, Table 3).

### 3.3. Duodenal and Hepatic Gene Expression of Related Proteins

**Figure 1:**
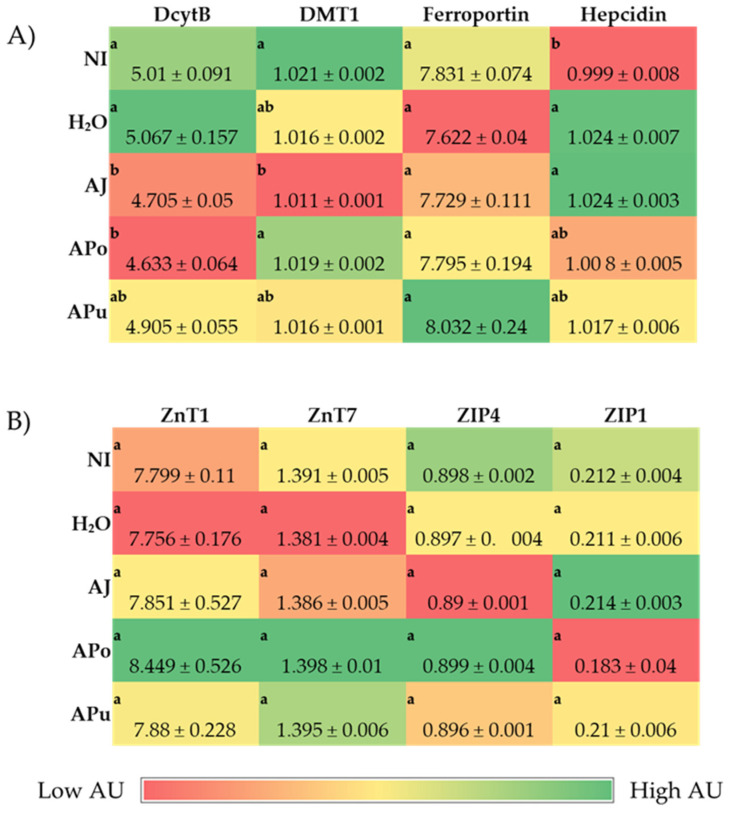
Effect of intra-amniotic administration of apple fraction soluble extracts on iron (**A**) and zinc (**B**) intestinal and liver (Hepcidin) gene expression. Values are the means ± SEM, *n* = 5. ^a, b^ Per gene, treatment groups not indicated by the same letter in the same column are significantly different (*p* < 0.05) by Duncan’s post-hoc test. DcytB, Duodenal cytochrome B; DMT1, Divalent metal transporter 1; Zinc transporters: ZnT1, ZnT7, ZIP4, ZIP1; AU, Arbitrary units.

**Figure 2:**
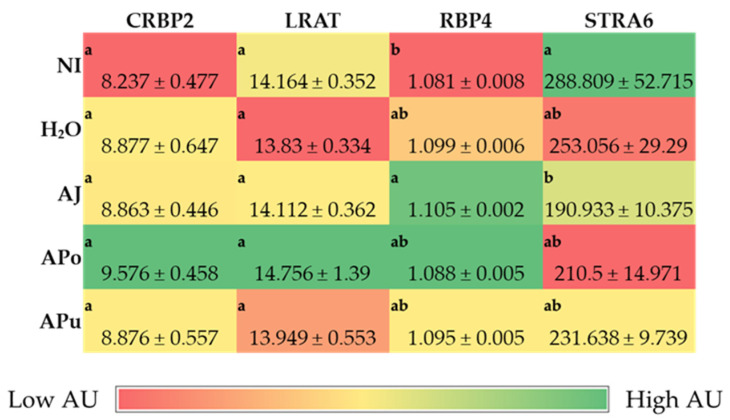
Effect of intra-amniotic administration of apple fraction soluble extracts on vitamin A intestinal (CRBP2 and LRAT) gene expression and hepatic (RBP4 and STRA6) metabolic proteins. Values are the means ± SEM, *n* = 5. ^a, b^ Treatment groups not indicated by the same letter in the same column are significantly different (*p* < 0.05) by Duncan’s post-hoc test. CRBP2, Cellular retinol-binding protein 2; LRAT, Lecithin: Retinol Acyltransferase; RBP4, Retinol binding protein 4; STRA6, Stimulated by Retinoic acid 6; AU, Arbitrary units.

**Figure 3:**
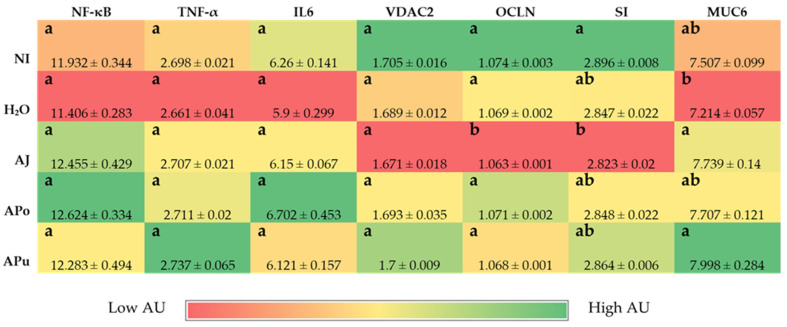
Effect of intra-amniotic administration of apple fraction soluble extracts on inflammatory and functional intestinal protein gene expression. Values are the means ± SEM, *n* = 5. ^a, b^ Treatment groups not indicated by the same letter in the same column are significantly different (*p* < 0.05) by Duncan’s post-hoc test. NF-kB, Nuclear factor kappa beta; TNF-α, Tumor necrosis factor-alpha; IL6, Interleukin 6, VDAC2, Voltage-dependent anion channel 2; OCLN, Occludin; SI, Sucrase isomaltase; MUC6, Mucin 6; AU, Arbitrary units.

Figure 1, Figure 2 and Figure 3 depict the gene expression of proteins relevant to micronutrients (iron, zinc, and vitamin A) metabolism, immune response, and functionality.

#### 3.3.1. Iron and Zinc-Related Proteins

DcytB expression in treatment groups AJ and Apo were downregulated (*p* < 0.05) compared to the NI and H_2_O controls (Figure 1). AJ reduced (*p* < 0.05) gene expression of DMT1 relative to the NI control, but no difference was observed relative to the H_2_O control. The apple groups did not alter (*p* < 0.05) the gene expression of ferroportin compared to the NI and H_2_O groups. Hepcidin, an iron-related protein located in the liver, was upregulated by the AJ treatment relative to the NI control. APo and APu were not significantly altered compared to NI and H_2_O controls.

The administration of apple juice, pomace, and pulp extractions did not alter (*p* < 0.05) the gene expression of zinc-related proteins at the brush border membrane relative to the NI and H_2_O controls.

#### 3.3.2. Vitamin A-Related Proteins

No significant differences (*p* < 0.05) were observed in the gene expression of CRBP2 and LRAT between the treatment and control groups (Figure 2). AJ had the greatest (*p* < 0.05) expression of RBP4 compared to the NI control, which had the lowest (*p* < 0.05) expression of RBP4, however, there were no differences (*p* < 0.05) between the H_2_O control and the apple treatment groups. 

#### 3.3.3. Inflammatory and Functionality-Related Proteins

The gene expression of proteins related to inflammation—NF-κB, TNF-α, and IL6—was not significantly different (*p* < 0.05) between the control and experimental groups (Figure 3). Further, gene expression of functional proteins VDAC2, SI, and MUC6 were not significantly (*p* < 0.05) different between treatment and control groups. However, the gene expression of OCLN was significantly (*p* < 0.05) lowered in the AJ group compared to all other treatment groups.

### 3.4. Morphometric Analysis

Groups APo and APu have significantly (*p* < 0.05) greater villi surface area compared to both control and experimental groups (Table 4). Crypt depth is significantly shortest (*p* < 0.05) in APu, APo, and AJ, respectively, related to both controls.

The intra-amniotic administration of AJ increased (*p* < 0.05) goblet cell diameter within the villi relative to the other experimental groups and H_2_O control, but not the NI control (Table 5). Goblet cell diameter within the crypts was significantly (*p* < 0.05) lower in the APo group. Further, the count of goblet cells within the intestinal crypts was highest (*p* < 0.05) in APu relative to the other treatment and control groups. The abundance of acidic goblet cells per unit area was more significant (*p* < 0.05) in the APu treatment compared to the other apple fraction treatments and NI and H_2_O controls. Neutral goblet cell abundance was significantly (*p* < 0.05) lowered in all apple fraction treatment groups relative to the H_2_O control but was similar to the NI control. Mixed goblet cells were most numerous (*p* < 0.05) in APu relative to the APo, NI, and H_2_O groups. 

With regard to the morphometric measurements of Paneth cells within the crypt, APo and APu groups had the greatest (*p* < 0.05) crypt Paneth cell count per unit area, respectively (Table 6). The H_2_O group had the lowest (*p* < 0.05) observed Paneth cell count per unit area relative to all other groups, yet had the greatest Paneth cell diameter.

### 3.5. Microbial Analysis

**Figure 4:**
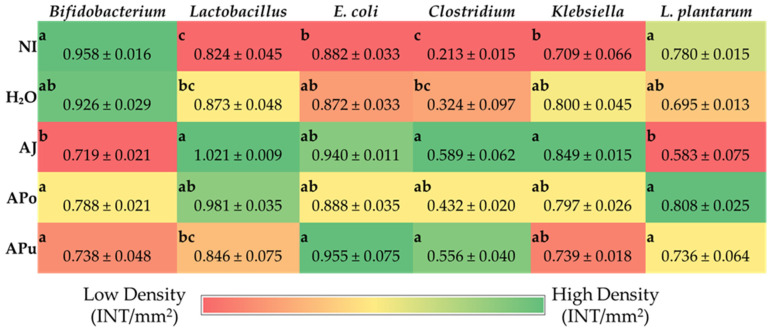
Effect of intra-amniotic administration of apple fraction soluble extracts on genera- and species-level bacterial populations from lower intestine contents on the day of hatch. Values are the means ± SEM, *n* = 5. ^a–c^ Treatment groups not indicated by the same letter in the same column are significantly different (*p* < 0.05) by Duncan’s post-hoc test.

The AJ group reduced the relative abundance of Bifidobacterium (*p* < 0.05) compared to the control and experimental groups (Figure 4). Furthermore, the greatest increase (*p* < 0.05) of *Lactobacillus* abundance relative to NI and water-only control groups occurred in the AJ group, while APo also increased (*p* < 0.05) *Lactobacillus* abundance relative only to the water injection control. All apple treatment groups have an increased (*p* < 0.05) abundance of *Clostridium* relative to the NI control, whereas only AJ and APu abundance is significantly higher than both controls. No differences were observed between the apple treatments relative to all controls for *Klebsiella* and *L. plantarum* microbial abundance.

## 4. Discussion

The Empire apple variety is a cross between McIntosh (*Malus domestica* “McIntosh”) and Red Delicious (*Malus domestica* “Red Delicious”) cultivars and is native to New York state [52]. According to the United States Apple Association, Empire apples are among the top-produced apples in the nation [6,53]. Here, we have investigated the effects of Empire apple juice (AJ), pomace (APo), and pulp (APu) extracts via intra-amniotic administration on micronutrient absorption, intestinal immune response, gut morphology, and cecal bacterial populations. To our knowledge, this is the first study to examine such physiological effects of the Empire apple cultivar. 

Body weight, blood glucose, and glycogen content (Table 3) did not differ throughout the treatment and control groups. As apples contain select macro- and micronutrients, consumption of this fruit and weight gain prevention have been studied in previous animal trials [54]. While Cho et al. (2013) reported apple pomace and juice supplementation reduced (*p* < 0.05) body weight gain in Sprague-Dawley rats [55], and Samout et al. (2016) found apple pectin to exert anti-obesity effects in Wistar rats [56], changes in body weight did not occur in our study. We hypothesize that this may be the case as treatment groups were a single dose in a naïve system, while the aforementioned studies utilized overweight models over a prolonged period. 

The intra-amniotic administration of apple juice and pomace extracts reduced the gene expression of DcytB reductase relative to the controls (Figure 1A). Located in the proximal duodenum, DcytB functions to reduce ferric dietary iron to the bioavailable form (Fe^2+^) for uptake into the enterocyte [57]. No significant changes occurred in the remaining iron metabolism proteins (DMT1, Ferroportin, and Hepcidin). Nonetheless, the reduced expression of DcytB potentially suggests an improvement in iron absorption into the enterocyte [58,59]. Shah et al. (2003) reported that apple juice enhances iron bioavailability in American children of 3 to 6 years of age [60]. The soluble apple fractions did not enhance zinc transporter proteins and vitamin A metabolism proteins, yet these results revealed no adverse effects of a single-dose administration. We expected to observe an anti-inflammatory effect of the apple treatments due to the naturally occurring phenolics in apples (represented in Table 2), which possess antioxidative properties (e.g., chlorogenic acid, quercetin glycosides, catechin) [21,22,23]. However, Figure 3 reveals no effect of the apple treatments on inflammatory cytokine expression (NF-κB, TNF-α, IL6). Given the reported anti-inflammatory potential of apples [10,18,19,20], the lack of agreement with this study’s results can be attributed to the short exposure time and concentrations administered. Conversely, our results reveal that apple pomace extract did not stimulate a negative intestinal immune response. Apple seeds are known to generate toxic cyanogenic glycosides upon grinding and have been a factor of concern when upscaling apple pomace for consumption. In a recent study, using Fisher rats, Ravn-Haren et al. investigated the effects of a different cultivar (Shampion) apple pomace with and without seeds [61]. It was reported that apple pomace, regardless of seed content, did not elevate alanine aminotransferase, a liver toxicity biomarker [61].

Intestinal barrier integrity is vital to gastrointestinal functionality and health, and tight junction proteins play a crucial role in maintaining the luminal structure [62,63]. Located in luminal epithelial cells, tight junctions, such as claudin and occludin regulate the permeability of ions, water, and macronutrients [64,65,66]. Expression of the tight junction protein occludin (OCLN) was significantly reduced by apple juice administration (Figure 3), which suggests an increase in epithelial permeability. Apple juice is known to naturally contain high amounts of simple sugars such as glucose and fructose [67,68]. High-sugar diets have reportedly increased intestinal barrier permeability, although the direct mechanism is unclear [69]. One proposed mechanism is through the intestinal microbiome, as diets rich in simple sugars have been linked to disrupting the balance of gut microbes, causing dysbiosis [69]. Dysbiosis can be characterized by an increase in the *Firmicutes*/*Bacteroidetes* ratio [69,70,71]. AJ increased the abundance of *Clostridium* and *Klebsiella*, as depicted in Figure 4. The *Clostridium* genus comprises commensal bacteria within the *Firmicutes* phylum that can exert pathogenic effects under dysbiosis conditions. Essentially, the overgrowth of opportunistic species within the *Clostridium* and *Klebsiella* may lead to the degradation of the intestinal barrier [72,73,74] or render severe infection [75]. Conversely, while the beneficial bacteria *Bifidobacterium* decreased abundance in AJ, *Lactobacillus* increased abundance. *Lactobacillus* is a lactic acid-producing bacteria within the *Firmicutes* phylum; thus, our results suggest a possible selective stimulation of *Firmicute* proliferation by AJ. 

Figure 4 reveals the increased abundance of *Clostridium* within APo and APu relative to the NI control. According to our results in Figure 3 and Table 4, Table 5 and Table 6, it is possible that the *Clostridium* genera exerted a beneficial effect as an induced effect on intestinal permeability and inflammatory cytokine expression was not observed. Therefore, we hypothesize that valuable species of *Clostridia* were increased. *Clostridium* is an SCFA-producing genus that has been reported to grow in abundance through pectin fermentation [76,77,78]. APo and APu had the greatest amount of non-fiber carbohydrates (including pectin) (Table 2), villi surface area (Table 4), and acidic goblet cells within the crypt (Table 5). A previous study reported that apple-derived pectin-fed rats increased *Clostridium* species abundance four-fold, whilst also increasing butyrate levels [79]. Butyrate is a short-chain fatty acid produced upon carbohydrate fermentation by *Clostridium* that can lower intestinal pH [78], which could explain the increased count of acidic goblet cells for APo and APu. Dufourny et al. (2021) previously assessed the effects of apple pomace on intestinal morphology and microbiota in weaned piglets. They found the pomace to increase *Clostridia* abundance and duodenal and ileal villi length [80]. Our results agree with the significant findings of this study. This further establishes the role of apple pomace to modulate Clostridia groups in both animal models which leads to improvements in gut health and intestinal homeostasis.

Shortened crypt depth was observed in all apple treatment groups (Table 4). Shortened crypt depths are morphological evidence for improved intestinal health as it suggests a slower intestinal epithelial cell turnover rate, allowing sufficient time for enterocytes to differentiate and function at capacity [81,82,83]. Within intestinal crypts, goblet cells play a role in maintaining the gut epithelial layer as they secrete mucus and mucin glycoproteins that function as a protective layer along the intestinal lumen [84]. A previous study found apple polysaccharide isolated from Fuji apple pomace to stimulate the enhancement of gut epithelial integrity by goblet cell autophagy [85]. Our study observed that APu and APo had the greatest crypt goblet cell count per unit area, respectively. In addition, APu had a significantly lower crypt goblet cell diameter. This finding likely suggests lower mucus content since intestinal crypt goblet cells only secrete mucus upon stimulation [84] Our findings suggest that the Empire apple has a level of effect on goblet cells located in the crypt. However, further studies should be completed to elucidate the potential impact. Moreover, Paneth cells are in the small intestinal crypts and function in the gut immunological response—secreting antimicrobial peptides and immunomodulating proteins to maintain intestinal homeostasis [86,87,88]. Crypt Paneth cell count per unit area was greatest (*p* < 0.05) in APo, APu, and AJ, respectively, compared to H_2_O control (Table 6). Yet, the Paneth cell diameter of APo treatment was similar (*p* > 0.05) to APu and AJ, and lower (*p* > 0.05) than the H_2_O control. Based on these observations and a recent review that summarized the impacts of dietary fiber on host gastrointestinal immune response, it seems that the Empire apple may stimulate Paneth cell function and improve intestinal immune response [89].

## 5. Conclusions

The intra-amniotic administration of Empire apple soluble extracts from various fractions was completed in this study. The data suggests that each apple fraction can alter duodenal brush border membrane functionality, morphology, and the cecal microbial populations. More specifically, the potential health benefits of apple pomace are revealed in this study, evident by reducing iron metabolism protein gene expression (DcytB), increasing villi surface area and decreasing crypt depth, increasing Paneth cell count per intestinal crypts, and increasing potentially beneficial gut bacteria (*Clostridium* spp.). Additional long-term studies should be completed to further establish potential health benefits.

## Figures and Tables

**Table 1:** DNA primer sequences used in this study.

Analyte	Forward Primer (5′-3′)	Reverse Primer (5′-3′)	Base Pair	GI Identifier
Iron Metabolism				
DcytB	CATGTGCATTCTCTTCCAAAGTC	CTCCTTGGTGACCGCATTAT	103	20380692
DMT1	TTGATTCAGAGCCTCCCATTAG	GCGAGGAGTAGGCTTGTATTT	101	206597489
Ferroportin	CTCAGCAATCACTGGCATCA	ACTGGGCAACTCCAGAAATAAG	98	423984
Hepcidin	AGACGACAATGCAGACTAACC	CTGCAGCAATCCCACATTTC	132	SAMN08056490
Zinc Metabolism				
ZnT1	GGTAACAGAGCTGCCTTAACT	GGTAACAGAGCTGCCTTAACT	105	54109718
ZnT7	GGAAGATGTCAGGATGGTTCA	CGAAGGACAAATTGAGGCAAAG	87	56555152
ZIP4	TCTCCTTAGCAGACAATTGAG	GTGACAAACAAGTAGGCGAAAC	95	107050877
ZIP1	TGCCTCAGTTTCCCTCAC	GGCTCTTAAGGGCACTTCT	144	121112053
Vitamin A Metabolism				
CRBP2	GGCTACATGGTTGCACTAGACA	AACCACCCGGTTATCGAGTC	195	NM_001277417.1
LRAT	GATTTTGCCTATGGCGGCAG	TTGTCGGTCTGGAAGCTGAC	197	XM_420371.7
RBP4	TGCCACCAACACAGAACTCTC	CTTTGAAGCTGCTCACACGG	149	NM_205238.2
STRA6	GTGCGCTGAACTTTGTCTGC	TTCTTCCTGCTCCCGACCT	116	NM_001293202.2
Inflammatory Response				
NF-κB	CACAGCTGGAGGGAAGTAAAT	TTGAGTAAGGAAGTGAGGTTGAG	100	2130627
TNF-α	GACAGCCTATGCCAACAAGTA	TTACAGGAAGGGCAACTCATC	109	53854909
IL6	ACCTCATCCTCCGAGACTTTA	GCACTGAAACTCCTGGTCTT	105	302315692
Brush Border Membrane Functionality				
VDAC2	CAGCACTCGCTTTGGAATTG	GTGTAACCCACTCCAACTAGAC	99	395498
OCLN	GTCTGTGGGTTCCTCATCGT	GTTCTTCACCCACTCCTCCA	124	396026
SI	CCAGCAATGCCAGCATATTG	CGGTTTCTCCTTACCACTTCTT	95	2246388
MUC6	CCAACTTGCAGTGTTCCAAAG	CTGACAGTGTAGAGCAAGTACAG	106	XM_015286750.1
18S rRNA	GCAAGACGAACTAAAGCGAAAG	TCGGAACTACGACGGTATCT	100	7262899

DcytB, Duodenal cytochrome B; DMT1, Divalent metal transport 1; ZnT1, Zinc transporter 1; ZnT7, Zinc transporter 7; CRBP2, Cellular retinol-binding protein 2; LRAT, Lecithin; Retinol Acyltransferase; RBP4, Retinol binding protein 4; STRA6, Stimulated by Retinoic aid 6; NF-kB, Nuclear factor kappa beta; TNF-α, Tumor necrosis factor-alpha; IL6, Interleukin 6; VDAC2, Voltage-dependent anion channel 2; OCLN, Occludin; SI, Sucrase isomaltase; MUC6, Mucin 6.

**Table 2:** Total polyphenolic (mg/g GAE) and fiber content of apple products.

Sample	TPC (mg/g GAE)	ADF (%/DM)	NDF (%/DM)	NFC (%/DM)
Pomace	0.834 ± 0.059 ^b^	22.6	25.6	62
Juice	0.300 ± 0.029 ^c^	NA	NA	NA
Pulp	1.57 ± 0.074 ^a^	6.7	7.9	85.4

Values are the means ± SEM, ^a–c^ Treatment groups not indicated by the same letter in the same column are significantly different (*p* < 0.05) by Duncan’s post-hoc test. ADF: cellulose, lignin, and insoluble minerals; NDF: cellulose, lignin, insoluble minerals, and hemicellulose; NFC: sugars, starches, organic acids, and pectin.; GAE: gallic acid equivalents; DM: dry matter; NA: not applicable.

**Table 3:** Effect of the intra-amniotic administration of apple fraction soluble extracts on body weight (g), blood glucose (mg/dL), and glycogen (mg/g).

Treatment Group	Body Weight (g)	Blood Glucose (mg/dL)	Glycogen (mg/g)
NI	40.83 ± 1.24 ^a^	254.11 ± 23.83 ^a^	0.396 ± 0.101 ^a^
H_2_O	38.29 ± 4.31 ^a^	234.4 ± 11.16 ^a^	0.294 ± 0.093 ^a^
AJ	40 ± 0.99 ^a^	225.5 ± 11.43 ^a^	0.431 ± 0.092 ^a^
APo	35.7 ± 0.67 ^a^	230.56 ± 21.28 ^a^	0.271 ± 0.054 ^a^
APu	36.44 ± 0.85 ^a^	205.5 ± 32.05 ^a^	0.442 ± 0.077 ^a^

Values are the means ± SEM, *n* = 5. ^a^ Treatment groups not indicated by the same letter in the same column are significantly different (*p* < 0.05) by Duncan’s post-hoc test.

**Table 4:** Effect of the intra-amniotic administration of apple fraction soluble extracts on villi surface area and crypt depth.

Treatment Group	Villi Surface Area (µm^2^)	Crypt Depth (µm)
NI	16,458.04 ± 771.84 ᵇ	22.1 ± 0.81 ᵃ
H_2_O	16,101.54 ± 383.07 ᵇ	21.93 ± 0.72 ᵃ
AJ	17,470.91 ± 444.08 ᵇ	14.45 ± 0.54 ᶜ
APo	23,116.65 ± 509.84 ᵃ	16.85 ± 0.79 ᵇ
APu	23,520.69 ± 739.04 ᵃ	17.38 ± 0.71 ᵇ

Values are the means ± SEM, *n* = 5. ^a–c^ Treatment groups not indicated by the same letter in the same column are significantly different (*p* < 0.05) by Duncan’s post-hoc test.

**Table 5:** Effect of the intra-amniotic administration of apple fraction soluble extracts on villi and crypt goblet cells.

Treatment Group	Villi Goblet Diameter (µm)	Crypt Goblet Diameter (µm)	Crypt Goblet Cell Number	Crypt Goblet Cell Number		
				Acidic	Neutral	Mixed
NI	3.48 ± 0.07 ^a^	3.01 ± 0.05 ^a^	7.01 ± 0.24 ^c^	5.79 ± 0.2 ^c^	0.02 ± 0.02 ^b^	1.21 ± 0.13 ^c^
H_2_O	3.17 ± 0.06 ^b^	2.89 ± 0.05 ^a^	8.55 ± 0.32 ^b^	6.92 ± 0.27 ^b^	0.13 ± 0.03 ^a^	1.51 ± 0.12 ^bc^
AJ	3.55 ± 0.07 ^a^	2.88 ± 0.05 ^a^	7.51 ± 0.26 ^c^	5.81 ± 0.22 ^c^	0.02 ± 0.01 ^b^	1.69 ± 0.11 ^ab^
APo	3.17 ± 0.06 ^b^	2.71 ± 0.06 ^b^	8.36 ± 0.27 ^b^	6.75 ± 0.23 ^b^	0.01 ± 0.01 ^b^	1.60 ± 0.10 ^b^
APu	3.08 ± 0.08 ^b^	2.84 ± 0.05 ^a^	9.14 ± 0.31 ^a^	7.35 ± 0.26 ^a^	0.00 ± 0.00 ^b^	1.79 ± 0.11 ^a^

Values are the means ± SEM, *n* = 5. ^a–c^ Treatment groups not indicated by the same letter in the same column are significantly different (*p* < 0.05) by Duncan’s post-hoc test.

**Table 6:** Effect of the intra-amniotic administration of apple fraction soluble extracts on Paneth cells.

Treatment Group	Crypt Paneth Cell Number	Paneth Cell Diameter (µm)
NI	1.22 ± 0.03 ^c^	1.37 ± 0.02 ^c^
H_2_O	1.04 ± 0.01 ᵈ	1.5 ± 0.02 ᵃ
AJ	1.31 ± 0.04 ^bc^	1.45 ± 0.02 ᵃᵇ
APo	1.44 ± 0.04 ᵃ	1.43 ± 0.02 ^b^
APu	1.38 ± 0.04 ^b^	1.45 ± 0.02 ᵃᵇ

Values are the means ± SEM, *n* = 5. ^a–d^ Treatment groups not indicated by the same letter in the same column are significantly different (*p* < 0.05) by Duncan’s post-hoc test.
